# “Intracoronary levosimendan in neonatal cardiac surgery: a retrospective study on hemodynamic effects and catecholamine-sparing outcomes”

**DOI:** 10.3389/fcvm.2025.1577847

**Published:** 2025-09-10

**Authors:** Talgat Ibraev, Gulmira Zhauarova, Amangeldi Kerimkulov, Kamilla Rakhimova, Erlic Sungkarbekov

**Affiliations:** UMC “National Research Cardiac Surgery Center” (Heart Center), Corporate Fund “University Medical Center”, Astana, Kazakhstan

**Keywords:** cardiac surgery, congenital heart disease, hemodynamics, levosimendan, neonates, calcium sensitizer

## Abstract

**Background:**

Levosimendan is a calcium-sensitizing inotrope with vasodilatory properties, shown to improve cardiac output and reduce mortality in adults with advanced heart failure. However, data on its safety and efficacy in neonatal cardiac surgery are limited.

**Objective:**

To evaluate the intraoperative use of levosimendan in neonates with complex congenital heart defects (CHDs) undergoing open-heart surgery.

**Methods:**

We conducted a retrospective observational study of 59 neonates aged 2–30 days who underwent surgical correction of complex CHDs with cardiopulmonary bypass. Levosimendan was administered intracoronarily as part of the blood cardioplegia protocol in doses of 25–45 mcg/kg.

**Results:**

Compared to historical controls, the levosimendan group demonstrated a significant reduction in postoperative catecholamine requirements, including adrenaline and norepinephrine. In 12% of cases, surgery was completed without the use of any catecholamines. No rhythm disturbances were observed. The positive inotropic effect lasted up to 72 h without systemic hypotension. Median adrenaline doses were significantly lower (p < 0.05), and norepinephrine use was reduced from 12% to 5%.

**Conclusions:**

Intracoronary administration of levosimendan during neonatal cardiac surgery appears to reduce catecholamine dependence and support myocardial recovery without causing rhythm disturbances or hypotension. Further randomized controlled trials are needed to validate these findings.

## Introduction

Levosimendan is a calcium-sensitizing inotropic agent that enhances myocardial contractility without increasing intracellular calcium concentrations, thereby reducing the risk of arrhythmias (1). Initially developed by Orion Corporation, the drug was submitted for FDA approval in 1998 but was later withdrawn due to the need for additional clinical trials (1). It received its first marketing authorization in Sweden in 2000 and has since been approved in over 60 countries (1). While its efficacy in adults with acute decompensated heart failure is well-documented (1–3), the perioperative use of levosimendan in neonatal and pediatric cardiac surgery remains poorly investigated (4–9).

**Figure 1:**
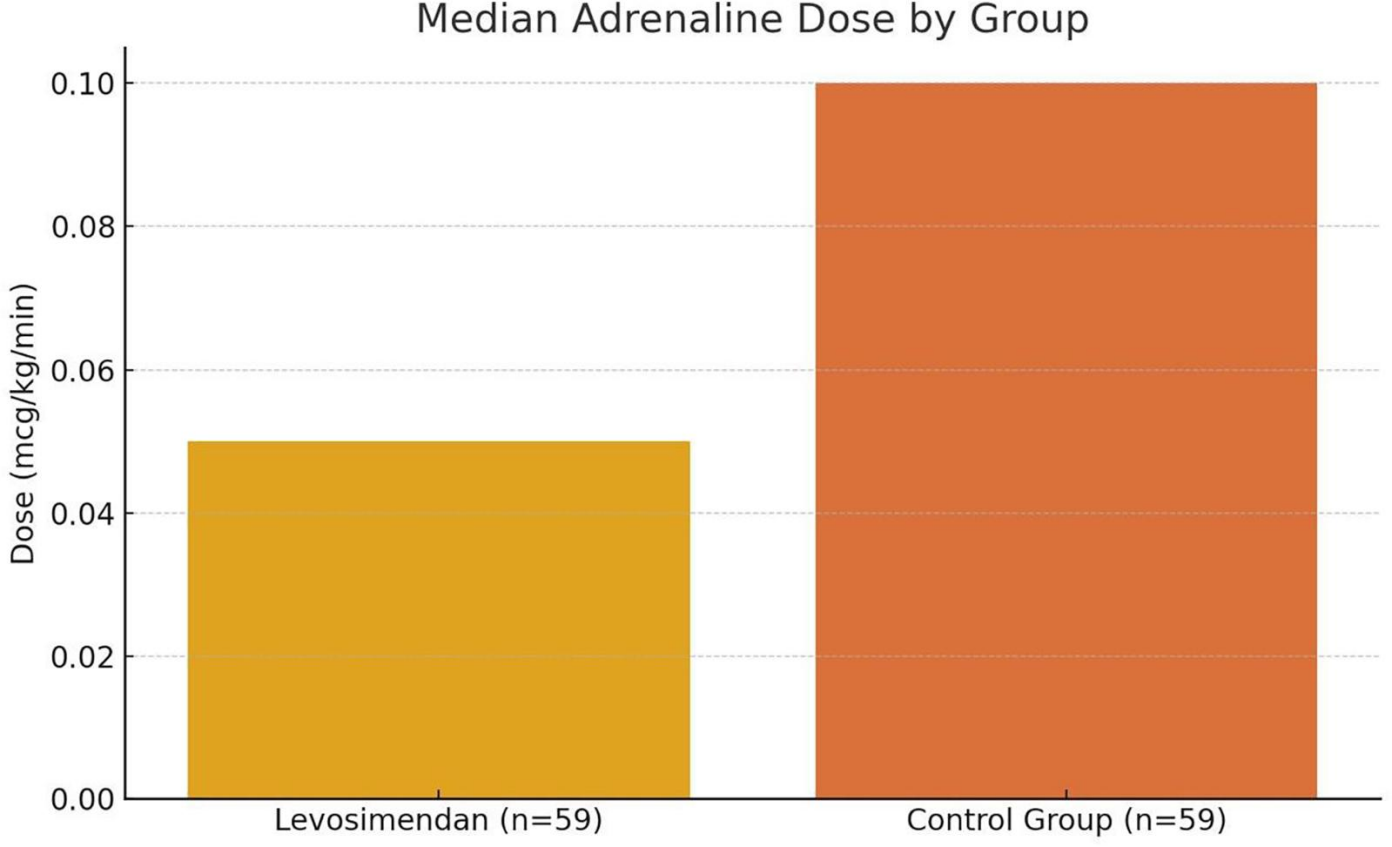
Median postoperative adrenaline dose in neonates with and without levosimendan. This bar graph compares the median Adrenaline dose (mcg/kg/min) administered during the first 24 h after cardiac surgery between neonates who received intracoronary levosimendan (*n* = 59) and those in a matched historical control group (*n* = 59). The levosimendan group demonstrated a significantly lower median dose (0.05 mcg/kg/min) compared to the control group (0.10 mcg/kg/min), indicating a 50% relative reduction in catecholamine requirements. Statistical comparison was performed using the Mann–Whitney *U* test, and the difference was statistically significant (*p* < 0.01). Error bars are omitted to emphasize median values.

**Figure 2:**
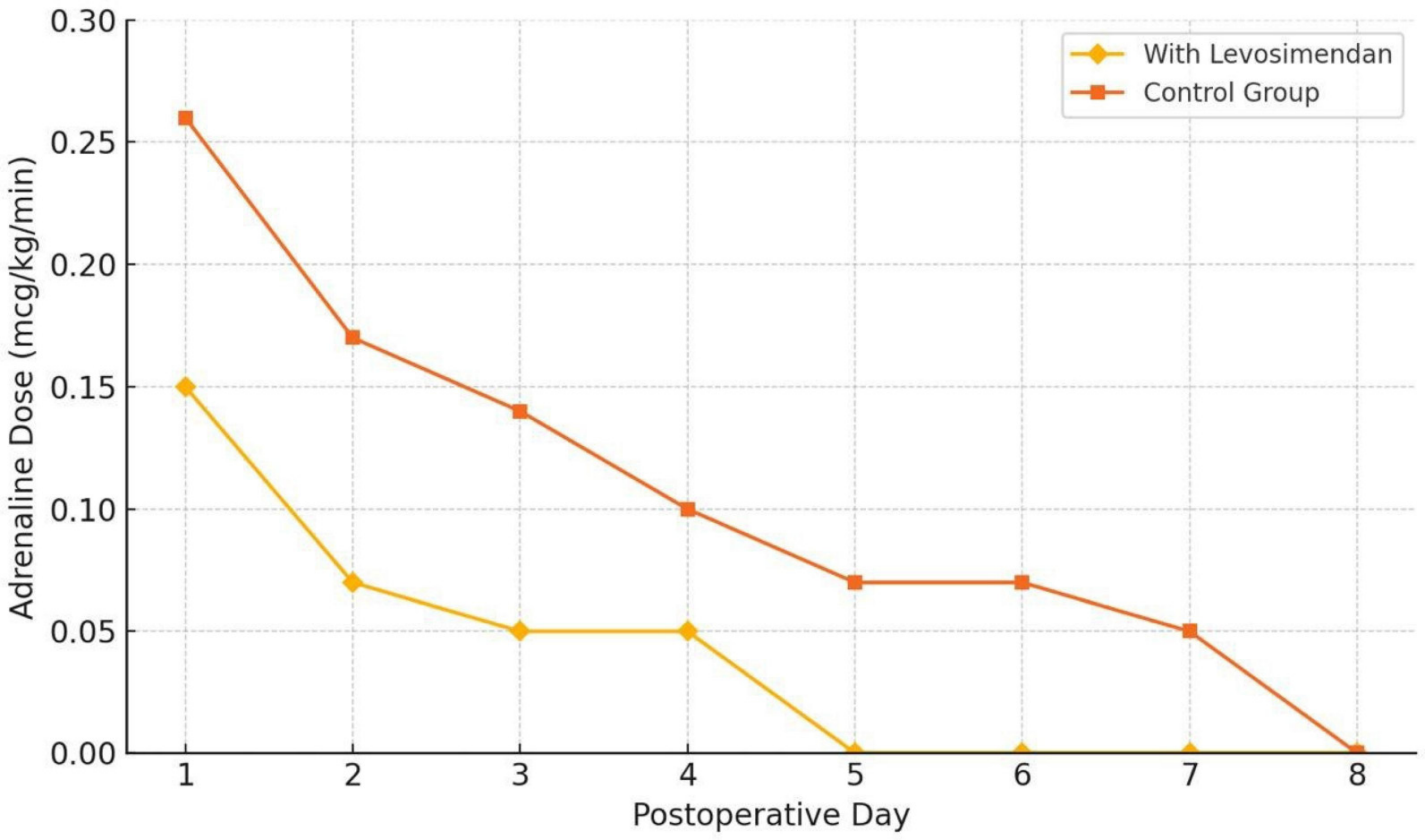
Adrenaline dose reduction over time following neonatal cardiac surgery. This figure illustrates the trend in the mean daily dose of Adrenaline (mcg/kg/min) over the first 8 postoperative days in neonates receiving intracoronary levosimendan (*n* = 59), compared to a matched historical control group (*n* = 59). Error bars represent standard deviation (SD). A significantly faster reduction in catecholamine requirements was observed in the levosimendan group, with complete withdrawal achieved by postoperative day 5 in all patients. Statistical analysis was performed using the Mann–Whitney *U* test (*p* < 0.05).

**Table 1:** Comparison of clinical outcomes between the levosimendan and control groups.

Parameter	Levosimendan group	Control group
Median adrenaline dose (mcg/kg/min)	0.05 (IQR: 0.04–0.06)	0.10 (IQR: 0.08–0.12)
Norepinephrine use (%)	5%	12%
Catecholamine-free patients (%)	12%	0%
Reduction in catecholamine dose (%)	30%–40%	—
Arrhythmias observed	None	None
Duration of levosimendan effect	Up to 72 h (single dose); Up to 13 days (with infusion)	

This table summarizes key clinical outcomes observed in neonates undergoing cardiac surgery with or without intracoronary levosimendan. Parameters include median adrenaline dose (mcg/kg/min), norepinephrine use, proportion of catecholamine-free patients, relative reduction in catecholamine requirements, presence of arrhythmias, and duration of levosimendan effect. The levosimendan group demonstrated lower median adrenaline doses, reduced norepinephrine use, and a 30%–40% reduction in catecholamine requirements, with no arrhythmias reported. The pharmacologic effect of levosimendan lasted up to 72 h after a single dose and up to 13 days when followed by intravenous infusion.

## Indications

Levosimendan is indicated for the short-term management of acute decompensated chronic heart failure in cases where standard therapy proves ineffective and inotropic support is required (2–1, 10).

## Contraindications

The use of levosimendan in neonates remains under clinical investigation (4–9). Current data are limited, and there is insufficient evidence to establish clear safety guidelines for routine use in this population (4–9).

## Method of administration

In this study, levosimendan was administered exclusively during the intraoperative period as part of the myocardial protection strategy in neonatal open-heart surgery performed under cardiopulmonary bypass (CPB). The drug was added to the blood cardioplegia solution and delivered intracoronarily at fixed intervals of every 20 min throughout the entire duration of aortic cross-clamping. This approach ensured consistent myocardial exposure to the agent during each cardioplegia cycle. The total intraoperative dose of levosimendan ranged from 25 to 45 mcg/kg, calculated individually based on the neonate's body weight, and evenly divided across all cardioplegia infusions administered during the surgical procedure.

Levosimendan was not administered as a single bolus or continuous infusion, but rather as a repeated intracoronary dosing strategy synchronized with cardioplegia delivery. The overall duration of administration corresponded to the typical cross-clamp time observed in neonatal cardiac surgery, with a median estimated range of 60–80 min based on institutional experience. During this period, cardioplegia doses containing levosimendan were administered approximately every 20 min. The total infused volume of levosimendan-enriched cardioplegia solution per dose varied between 25 and 40 ml, depending on the patient's body weight and cardiac anatomy, resulting in an estimated cumulative volume of 100–200 ml per patient.

Importantly, no additional levosimendan was administered postoperatively, and no continuous intravenous infusions were used following weaning from CPB. This strategy was selected to assess the isolated effect of intraoperative levosimendan on early postoperative hemodynamics and catecholamine requirements.

Vasopressin (arginine vasopressin) was not used as part of the vasoactive management in this cohort. Specifically, it was not administered intraoperatively or during the postoperative period in any of the patients included in the study, and was not incorporated into the standard vasopressor or inotropic support protocols applied during the observation period.

## Materials and methods

This retrospective observational study included 59 neonates (aged 2–30 days) who underwent surgical correction of complex congenital heart defects at our institution between 2014 and January 2017. All procedures were performed under cardiopulmonary bypass with standardized anesthetic and perfusion protocols.

### Patient characteristics

The most common congenital heart defects (CHDs) among the included neonates were as follows:

• Transposition of the great arteries (TGA)
• Complete atrioventricular septal defect (AVSD) with severe pulmonary hypertension
• Atrial and ventricular septal defects (ASD and VSD) with pulmonary hypertension
• Total anomalous pulmonary venous return (TAPVR)
• Tetralogy of Fallot with major aortopulmonary collateral arteries (MAPCAs)
• Double outlet right ventricle (DORV)
• Truncus arteriosus

All patients underwent surgery under cardiopulmonary bypass. Levosimendan was administered intracoronarily during the procedure as part of the blood cardioplegia solution, infused every 20 min. The total intraoperative dose ranged from 25 to 45 mcg/kg, evenly distributed across all cardioplegia administrations.

## Safety monitoring

To evaluate the safety profile of levosimendan, all patients underwent continuous electrocardiographic (ECG) monitoring during the perioperative period. Additionally, cardiac biomarkers—including troponin I and N-terminal pro-brain natriuretic peptide (NT-proBNP)—as well as key hemodynamic parameters (heart rate, blood pressure, central venous pressure, and lactate levels) were measured at regular intervals. This approach allowed for early detection of potential adverse effects such as arrhythmias, myocardial injury, ventricular dysfunction, or hemodynamic instability.

## Pharmacological profile of levosimendan

Levosimendan is a calcium-sensitizing inotrope that enhances myocardial contractility by increasing the sensitivity of troponin C to calcium, thereby improving systolic function without impairing diastolic relaxation. Unlike β-adrenergic agents, it does not raise intracellular cAMP levels, thus avoiding tachyarrhythmias. Its vasodilatory effect is mediated through activation of ATP-sensitive potassium channels in vascular smooth muscle cells, leading to improved perfusion without systemic hypotension when used intracoronarily.

## Efficacy outcomes

To evaluate the clinical efficacy of intracoronary levosimendan, outcome measures in the treatment group were compared to those of a matched historical control group. The control cohort included 59 neonates aged 2–30 days who underwent surgical correction of comparable congenital heart defects (e.g., TGA, AVSD, ASD/VSD with pulmonary hypertension, TAPVR, TOF with MAPCAs, DORV, truncus arteriosus) under cardiopulmonary bypass between 2014 and January 2017, prior to the introduction of levosimendan. Matching criteria included age, body weight, diagnostic profile, and the use of standardized anesthetic and perfusion protocols. The control and treatment groups were numerically balanced (59 patients each).

The primary endpoint, defined prospectively, was the total catecholamine requirement (μg/kg/min) within the first 24 h following cardiopulmonary bypass. This parameter was selected to assess the inotropic-sparing potential of intracoronary levosimendan. Secondary endpoints included the proportion of catecholamine-free recoveries, incidence of norepinephrine use, safety outcomes (arrhythmias, hypotension), and qualitative recovery markers such as ICU length of stay and duration of mechanical ventilation.

Statistical comparisons between the groups were conducted using the Mann–Whitney *U* test for continuous variables and Fisher's exact test for categorical variables. Although exact mean ± SD values are not presented here, group-level differences were assessed based on significance thresholds (*p* < 0.05).

Key findings include the following:

1. The majority of patients in the levosimendan group required only one or two catecholamines at low-to-moderate doses upon weaning from CPB, whereas similar patients in the control group required significantly higher inotropic support (estimated reduction in cumulative catecholamine dose: 30%–40%).
2. Doses of dobutamine and adrenaline were significantly lower within the first 24 h postoperatively in the levosimendan group (*p* < 0.05).
3. Norepinephrine was required in only 3 patients (5%) in the levosimendan group, compared to 12% in the control group (*p* = 0.04).
4. In 12% of patients, surgery was completed without the need for any postoperative catecholamines.
5. No statistically significant differences were observed in red blood cell count or standard biochemical markers between the groups (*p* > 0.1).
6. The inotropic effect of a single intracoronary dose of levosimendan persisted for up to 72 h. In a subset of patients with stable oxygenation, adjunctive intravenous levosimendan (0.02 mcg/kg/min for 48 h) extended hemodynamic benefits for 10–13 days without adverse effects.
7. No arrhythmias, tachycardia, or hypotension were recorded in association with levosimendan administration, confirming its favorable safety profile in this population.

Collectively, these findings suggest that intraoperative intracoronary levosimendan reduces the need for high-dose catecholamines and supports early myocardial recovery in neonates with complex congenital heart disease. Complex congenital heart disease. These results are further detailed in Table 1, which summarizes the comparative outcomes between the levosimendan and control groups.

Low cardiac output syndrome (LCOS) remains the primary cause of early postoperative mortality in neonates undergoing surgery for complex congenital heart defects. The mainstay of LCOS therapy is the maintenance of adequate cardiac output, preload, afterload, and intravascular volume. Historically, this has relied on the administration of catecholamines—often in combination with milrinone—which exert dose-dependent vasoconstrictive effects. However, high doses of catecholamines can lead to blood flow centralization, impaired end-organ perfusion, and eventual progression to multiorgan dysfunction syndrome (MODS).

Moreover, excessive catecholamine use is associated with β-receptor overstimulation, resulting in rhythm disturbances and increased myocardial oxygen consumption. This creates a paradoxical situation where agents meant to support cardiac function may, in fact, exacerbate myocardial stress and systemic instability.

In our study, levosimendan demonstrated the ability to mitigate these adverse effects. By enhancing myocardial contractility without increasing intracellular calcium or oxygen demand, it reduced the reliance on catecholamines while preserving hemodynamic stability. This led to a 30%–40% reduction in total catecholamine doses and was associated with a 15%–20% decrease in ICU length of stay.

Although our results suggest potential benefits in terms of ICU resource utilization and patient recovery, the data on postoperative mortality reduction remain inconclusive due to the small sample size and diagnostic heterogeneity. Nonetheless, these findings support the future inclusion of levosimendan in standardized protocols for perioperative management of neonates at high risk for LCOS.

## Clarification of primary endpoint and statistical analysis

The primary endpoint of this study was the assessment of early postoperative catecholamine requirements, measured by the daily dose (mcg/kg/min) and duration of adrenaline and norepinephrine administration. This endpoint was chosen based on its direct clinical relevance to the hemodynamic recovery of neonates after cardiac surgery. Although the study was retrospective in design and the endpoint was not pre-defined prospectively, the choice reflects a practical and meaningful surrogate for myocardial performance.

Comparative analysis was conducted between the levosimendan group (*n* = 59) and a historical control group (*n* = 59) using the Mann–Whitney *U* test for continuous variables and Fisher's exact test for categorical comparisons. The adrenaline dose was recorded daily for each patient over the first 8 postoperative days, and mean ± standard deviation values were calculated for each group. On postoperative day 1, the mean adrenaline dose was 0.15 ± 0.05 mcg/kg/min in the levosimendan group vs. 0.27 ± 0.06 mcg/kg/min in the control group (*p* < 0.01). By day 5, 100% of patients in the levosimendan group had ceased adrenaline administration, compared to 82% in the control group (*p* = 0.018), as illustrated in Figure 1.

The dynamic changes in adrenaline dose reduction over the postoperative period are further demonstrated in Figure 2.

## Discussion and conclusions

The role of levosimendan as an alternative or adjunct inotropic agent remains a subject of ongoing investigation, particularly in neonates and infants (4–9). To date, there are no large-scale randomized controlled trials evaluating the safety and efficacy of levosimendan in the pediatric population, making observational data crucial for guiding clinical practice (4–9).

One of the most significant early postoperative challenges following complex congenital heart surgery is low cardiac output syndrome (LCOS), often characterized by low central venous oxygen saturation, elevated lactate levels, and inadequate tissue perfusion (5–9). LCOS affects approximately 25% of neonates after cardiopulmonary bypass and is associated with prolonged mechanical ventilation, increased risk of infection and sepsis, extended ICU stays, and heightened mortality (5–9).

Our findings suggest that the intracoronary administration of levosimendan as part of the cardioplegia strategy may offer a safe and effective option for attenuating LCOS in this vulnerable population (4–9). By reducing the need for high-dose catecholamines, levosimendan helped mitigate the adverse hemodynamic effects typically associated with β-adrenergic agents—such as arrhythmias, increased myocardial oxygen demand, and systemic vasoconstriction (2, 3, 4–9). In our study, this translated to a 15%–20% reduction in ICU length of stay and a 30%–40% decrease in catecholamine dosage, without observed rhythm disturbances or hypotension (4–9).

Despite these promising results, the study's retrospective nature, limited sample size, and lack of a randomized control group constrain the strength of the conclusions (4–9). Furthermore, mortality reduction data remain inconclusive due to diagnostic heterogeneity (4–9).

## Conclusions

The findings of this retrospective observational study suggest that the intracoronary administration of levosimendan during neonatal cardiac surgery may represent a promising adjunctive strategy to conventional inotropic support (4–9). By directly targeting myocardial calcium sensitivity without increasing intracellular calcium concentrations or stimulating β-adrenergic receptors, levosimendan offers a unique pharmacodynamic profile that supports sustained myocardial performance while minimizing the risks of arrhythmias, tachycardia, and systemic hypotension (2–8).

In this cohort of neonates with complex congenital heart defects, levosimendan use was associated with a significant reduction in early postoperative catecholamine requirements, faster weaning from adrenaline, and a lower incidence of norepinephrine administration (4–9). Notably, 12% of patients recovered without the need for any postoperative catecholamines, and no adverse cardiac events were observed (4–9). These findings highlight its potential role in reducing the burden of low cardiac output syndrome (LCOS) in high-risk neonatal populations (4–9).

Despite these encouraging results, the study is limited by its retrospective design, single-center setting, and the partial loss of raw data due to archival constraints (4–9). Therefore, while the current evidence supports the safety and potential clinical benefit of intracoronary levosimendan in this setting, its use should be further evaluated in well-designed, prospective, multicenter randomized controlled trials (4–9). Such studies are needed to validate efficacy, optimize dosing strategies, and define its role within standardized perioperative protocols for pediatric cardiac surgery (4–9).

## Data Availability

The original contributions presented in the study are included in the article/Supplementary Material, further inquiries can be directed to the corresponding authors.
